# Information Geometry in Roegenian Economics

**DOI:** 10.3390/e24070932

**Published:** 2022-07-05

**Authors:** Constantin Udriste, Ionel Tevy

**Affiliations:** Department of Mathematics-Informatics, Faculty of Applied Sciences, University Politehnica of Bucharest, Splaiul Independentei 313, Sector 6, 060042 Bucharest, Romania; ionel.tevy@upb.ro

**Keywords:** statistical Roegenian economics, information geometry (ideal income, Van der Waals income), economic partition function, Fisher–Rao metric, scalar curvature, geodesics, 53B20, 60D99

## Abstract

We characterise the geometry of the statistical Roegenian manifold that arises from the equilibrium distribution of an income of noninteracting identical economic actors. The main results for ideal income are included in three subsections: partition function in distribution, scalar curvature, and geodesics. Although this system displays no phase transition, its analysis provides an enlightening contrast with the results of Van der Waals Income in Roegenian Economics, where we shall examine the geometry of the economic Van der Waals income, which does exhibit a “monetary policy as liquidity—income” transition. Here we focus on three subsections: canonical partition function, economic limit, and information geometry of the economic Van der Waals manifold.

## 1. Introduction

We have been inspired from information geometry in Thermodynamics (see [1]) to produce an information geometry in Roegenian economics [2,3,4,5,6,7,8,9,10,11,12,13,14,15,16,17].

For this, we used a part of Udriste team dictionary (a table that lists the words of a thermodynamic language and their correspondents in economics, associating “elements” that behave similarly; it is similar to a morphism—a structure-preserving map from one mathematical structure to another one of the same type). The dictionary part what interests us here is
    THERMODYNAMICS
        ECONOMICSU = internal energy…    G = growth potenţialT = temperature…    I = internal political stabilityS = entropy…    E = entropyP = pressure…    P = price level (inflation)V = volume…    Q = volume, structure, qualityM = total energy (mass)…    Y = national income (income)Q = electric charge…    I = total investmentW = mechanical work…    W = wealth of the systemQ = heat…    q = stock market*h* = Planck constant…    *h* = economic quantum

**Definition** **1.** *The economy based on rules similar to those in thermodynamics, via Udriste team dictionary (morphism), is called Roegenian economics.*

In Roegenian economics, *P* means price level, *I* means internal politics stability, *Q* means goods volume, and *Y* means national income.

Generally, we develop the vision of Nicholas Georgescu Roegen, which links the economic phenomena of entropy and other mathematical and physical elements of a significant economic nature. We will study the information geometry necessary in the case of aggregation of initially stable and independent economic subsystems in a functional entity, e.g., the architecture of the European Community Economy.

Although the system of ideal income, Section 2, displays no phase transition, our analysis provides an enlightening contrast with the results of Section 3 where we shall examine the geometry of the Van der Waals income, which does exhibit a “monetary policy as liquidity—income” transition. The topics of Section 2 are: partition function in P−I distribution, curvature for ideal income, and geodesics for ideal income. The topics of Section 3 include: state equation, canonical partition function, economic limit, and geometry of the economic Van der Waals manifold.

Generally, the information geometry approach [1,2,16,17,18,19,20] studies the differential geometric structure of statistical models. Refs. [1,18,21,22] helped us to complete our ideas on the geometry of statistical manifolds with economic significance. In fact, the geometrization of economic systems produces a surplus of readable information through geodesics, curvature, etc.

## 2. Ideal Income in Roegenian Economics

Let us characterise the geometry of the statistical Roegenian manifold that arises from the equilibrium distribution of an ideal income (see [16]) of noninteracting economic actors.

### 2.1. Partition Function in P−I Distribution

To elucidate the geometrical representation of ideal income systems in statistical Roegenian economics, we begin our analysis with a system of noninteracting identical economical actors in the absence of potential energy. Economically, this system corresponds to a classical ideal income immersed in an economic system of goods. As we shall show below, the Riemann curvature tensor field is 0 and the geodesic equations can be solved exactly.

Let *H* be the economic Hamiltonian (mimics “the free particle kinetic energy”),

$$H=\sum_{i=1}^{N}\;\frac{p_{i}^{2}}{2y},$$

where *y* means income, the same for each actor. We consider, in particular, a ”price level—internal politics stability” P−I distribution (see also known Boguslavski distribution in Thermodynamics) of the form

(1) $$p\left(H,Q|\alpha ,\beta \right)=\frac{\exp(-\beta H-\alpha Q)}{Z(\alpha ,\beta )}$$

defined on the phase space Γ of an economic system of goods, where *H* is the economic Hamiltonian, and *Q* is the goods volume. Here, the partition function Z(α,β) is determined by the phase-space and goods volume integral

(2) $$Z\left(\alpha ,\beta \right)=\frac{1}{N!h^{3N}}\int_{0}^{\infty }\left(\int_{\Gamma }\exp\left(-\beta H\right)\;dq\;dp\right)\;\exp\left(-\alpha Q\right)\;\;dQ,$$

where $\beta =\frac{1}{k_{B}I},\alpha =\frac{P}{k_{B}I}$, and *P* denotes the price level, *h* the economic quantum, and *N* the number of actors in the system.

Thus, we consider a closed system of noninteracting actors immersed in an economic system of goods at “inverse internal politics stability” $\frac{1}{I}$ and effective “price level” *P*. Since the system has fixed “internal politics stability” and “price level”, the system energy and the volume of goods fluctuate. In *I*-equilibrium, the distribution of these variables is determined by the Formula (1). For a real income, the constituent actors inevitably interact. Nevertheless, the ideal income represented by the distribution (1) adequately characterises the properties of a real income at high *I* or few actors in the system, where the effects of inter-actors’ interactions can be neglected.

Comparing (1) with exponential form of distributions, we observe that the economic potential is given by ψ(α,β)=lnZ(α,β). Therefore, to determine the Fisher–Rao metric, we must perform firstly the integration (2). Highlighting the fact that each *q*-integration in (2) gives the goods volume *Q* of the system, one obtains the partition function

(3) $$Z\left(\alpha ,\beta \right)={\left(\frac{2\pi y}{h^{2}\beta }\right)}^{\frac{3N}{2}}\;\;\alpha^{-(N+1)}.$$

This follows from the fact that

$$\int_{\Gamma }\exp\left(-\beta H\right)\;dq\;dp=\int_{q}\left(\int_{p}e^{-\beta \sum_{i=1}^{N}\frac{p_{i}^{2}}{2y}}\;\;\prod_{i=1}^{N}dp_{i}\right)\prod_{j=1}^{N}dq_{j}$$

is just a product of Gaussian integrals, and the identity

$$\frac{1}{N!}\int_{0}^{\infty }V^{n}e^{-\alpha Q}\;dQ=\alpha^{-(N+1)}$$

is true for α>0.

Note that the partition function z(β,Q) in the canonical ensemble is

$$z\left(\beta ,Q\right)=\frac{1}{N!h^{3}N}\int_{\Gamma }\exp\left(-\beta H\right)\;dq\;dp=\frac{1}{N!}{\left(\frac{2\pi y}{h^{2}\beta }\right)}^{\frac{3N}{2}}Q^{N},$$

from which one can calculate the Helmholtz free energy

$$F\left(\beta ,Q\right)=-k_{B}I\;\ln z\left(\beta ,Q\right)$$

and thus obtain the equation of state

$$P=-{\left(\frac{\partial F}{\partial Q}\right)}_{\beta }=N\;\;\frac{k_{B}I}{Q},$$

satisfied by a classical ideal income (see [16]).

### 2.2. Scalar Curvature for Ideal Income

The expression (3) for the partition function clearly shows that the Riemannian geometry of the statistical model M associated with the classical ideal income depends upon the number *N* of actors. Although finite size effects in small systems are sometimes of interest, here we are primarily concerned with the geometry that arises in the so-called economic limit N→∞. Thus, we consider the economic potential ψ(α,β) per actor in the economic limit, given by

$$\psi \left(\alpha ,\beta \right)=\lim_{N\to \infty }N^{-1}\ln Z\left(\alpha ,\beta \right)=\frac{3}{2}\left(\ln\frac{2\pi m}{h^{2}}-\ln\beta \right)-\ln\alpha .$$

The covariant components $g_{ij}$ of the Fisher–Rao Hessian metric [1,2] are

$$g_{11}=\frac{\partial^{2}\psi }{\partial \alpha^{2}},\;\;g_{12}=\frac{\partial^{2}\psi }{\partial \alpha \partial \beta },\;\;g_{22}=\frac{\partial^{2}\psi }{\partial \beta^{2}}$$

or explicitly

(4) $$g_{11}=\alpha^{-2},\;\;g_{12}=0,\;\;g_{22}=\frac{3}{2}\;\;\beta^{-2}.$$

It follows the contravariant components

(5) $$g^{11}=\alpha^{2},\;\;g^{12}=0,\;\;g^{22}=\frac{2}{3}\;\;\beta^{2}.$$

From this expression, we deduce the following.

**Proposition** **1.** *The Riemann tensor field of the statistical manifold M, associated with the classical ideal income, vanishes and thus the manifold is flat. Consequently, the scalar curvature also vanishes identically.*

**Proof.**  From the components of the metric (4), one can calculate the Christoffel symbols $\Gamma_{jk}^{i}$ and the components of the Riemann curvature tensor field $R_{ijkl}$ using the definitions.

Alternatively, to show that this manifold is flat, it suffices to display a change of coordinates which transforms the metric (5) into a Euclidean metric. Here, we adopt the latter approach because it permits a more expeditious solution. We recall that, under a coordinate transformation $x^{i}\to {\bar{x}}^{i}$, the metric of a Riemannian manifold transforms in the usual tensorial manner, so that the components of the inverse metric in the new coordinate system are determined by the formula

$${\bar{g}}^{kl}=g^{ij}\;\;\frac{\partial {\bar{x}}^{k}}{\partial x^{i}}\frac{\partial {\bar{x}}^{l}}{\partial x^{j}}.$$

Now, we fix the following coordinate transformation:

$$\alpha \to \bar{\alpha }=\ln\alpha ,\;\;\beta \to \bar{\beta }=\sqrt{\frac{3}{2}}\ln\beta .$$

A straightforward calculation then shows that the components of the inverse metric in the $\left(\bar{\alpha },\bar{\beta }\right)$ coordinate system are

$${\bar{g}}^{11}=1,\;{\bar{g}}^{12}=0,\;{\bar{g}}^{22}=1,$$

and thus the manifold is indeed flat, and the geodesics are straight lines. □

### 2.3. Geodesics for Ideal Income

Recall that *P* means price level, and *I* means internal politics stability. The geodesics of the statistical manifold M include the derivation of P−I relation at a constant level of goods production regarded as supply (corresponding to adiabatic transformations).

**Proposition** **2.** *The geodesic curves on the statistical manifold M associated with the classical ideal income are given by*

$$\frac{P}{P_{0}}={\left(\frac{k_{B}I}{k_{B}I_{0}}\right)}^{1+c},$$

*where $P_{0},\;I_{0}$, and c are integration constants. In particular, the geodesics include the constant level of good curves of state for the ideal income, corresponding to the choice $c=-C_{Q}/Nk_{B}$, where $C_{Q}$ is an economic capacity.*

**Proof.**  The geodesic equations for the variables α and β assume identical forms, i.e.,

$$\frac{d^{2}x}{ds^{2}}+\frac{1}{x}{\left(\frac{dx}{ds}\right)}^{2}=0,\;\;\mathrm{for}\;\;x=\alpha ,\beta .$$

This can be rewritten as

$$\frac{d}{ds}\ln\frac{dx}{ds}=\frac{d}{ds}\ln x,$$

from which we see that the general solution is $x\left(s\right)=c_{1}e^{c_{2}s}$, where $c_{1}$, $c_{2}$ are arbitrary constants. Thus, we obtain

$$\frac{P}{k_{B}I}=c_{1}e^{c_{0}s},\;\;\mathrm{and}\;\;\frac{1}{k_{B}I}=c_{3}e^{c_{2}s}$$

as the general solution to the geodesic equations. Eliminating the parameter *s*, we obtain the formula in proposition written in the form

$$P=\frac{c_{1}}{c_{3}^{c_{0}/c_{2}}}{\left(k_{B}I\right)}^{1-c_{0}/c_{2}}.$$

Setting s=0, we find $c_{1}=P_{0}/k_{B}I_{0}$ and $c_{3}=1/k_{B}I_{0}$, which yields at once the expression in the Proposition. □

## 3. Van Der Waals Income in Roegenian Economics

The geometry of the statistical manifold M changes considerably if the economic actors interact. In particular, if the system exhibits a phase transition, then the scalar curvature tends to become singular at the transition point. This property seems to be universal and appears in many systems exhibiting critical phenomena. The Van der Waals income model is not only of economic interest, but also illustrates many of the universal geometrical features of the associated manifold of equilibrium states.

### 3.1. Van Der Waals State Equation

Let *P* be the price level, *Q* be the volume of goods, and *I* be internal politics stability. Here, we shall extend the model to include economic actors’ interactions, which leads to the economic Van der Waals equation of state (see also [16])

(6) $$\left(P+a\;\;\frac{N^{2}}{Q^{2}}\right)\left(Q-bN\right)=Nk_{B}I,$$

where *N* is the total number of economic actors and a,b are constants determined by the properties of each actor. The “monetary policies as liquidity or consumption—income” transition occurs at the critical point where price level *P*, volume of goods *Q*, and the internal politics stability *I* assume the values

$$P_{c}=\frac{a}{27b^{2}},\;\;Q_{c}=3bN,\;\;I_{c}=\frac{8a}{27k_{B}b}.$$

### 3.2. Canonical Partition Function

The economic equation of state (6) is similar to the Van der Waals equation in thermodynamics [23]. However, it can also be derived analytically from the canonical partition function associated with an empirically postulated inter-actors potential. Assume that the interaction energy between a pair of economic actors, separated by an “economic distance” *r*, is given by an economic potential (similar to the Lennard–Jones potential in thermodynamics)

$$\phi \left(r\right)=4\phi_{0}\left[{\left(\frac{d}{r}\right)}^{12}-{\left(\frac{d}{r}\right)}^{6}\right]=\phi_{0}{\left(\frac{r_{0}}{r}\right)}^{12}-2\phi_{0}{\left(\frac{r_{0}}{r}\right)}^{6},$$

where $r_{0}=2^{1/6}d$, and *d* is a parameter which can be regarded as the radius of the economic actor. Clearly, ϕ(d)=0 and ϕ(r) assume its minimum value $-\phi_{0}$ at $r=r_{0}$. As we see in the table of variations,

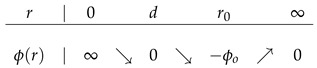

This inter-actor potential energy gives rise to a weak long-range attractive economic force and a strong short-range repulsive economic force between each pair of actors. The canonical partition function can thus be written as

$$z\left(\beta ,Q\right)=\frac{1}{N!h^{3N}}\prod_{i=1}^{N}\;\;\int d^{3}p_{i}\int d^{3}r_{i}\;\;\exp\left(-\beta \sum_{i=1}^{N}\frac{p_{i}^{2}}{2y}-\beta \sum_{\left(ij\right)}\phi_{ij}\right),$$

where $\phi_{ij}=\phi \left(r_{ij}\right)$, with $r_{ij}$ denoting the “economic distance” between the *i*-th and *j*-th actors, and *y* means income, the same for each economic actor. Thus, the canonical partition function can be expressed as a product

$$z\left(\beta ,Q\right)=z_{0}\left(\beta ,Q\right)\;\;q\left(\beta ,Q\right),$$

where $z_{0}\left(\beta ,Q\right)$ is the canonical partition function for the ideal income (see Section 2) and

(7) $$q\left(\beta ,Q\right)=\frac{1}{Q^{N}}\int d^{3}r_{1}\cdots \int d^{3}r_{N}\;\;\exp\left(-\beta \sum_{\left(ij\right)}\phi_{ij}\right)$$

is the contribution from the interaction energy. Now, as an approximation to the economic Lennard-Jones potential, we assume that $\exp(-\beta \phi_{ij})=0$ for $r_{ij}<d$. In other words, we regard the actors as "hard spheres" of radius *d*, which cannot overlap. As a consequence, the overlapping region can be removed from the range of the volume of goods integration (7). Defining the so-called Mayer function $f_{ij}=f\left(r_{ij}\right)$ by $f_{ij}=\exp\left(-\beta \phi_{ij}\right)-1$, we rewrite the integral (7) as

(8) $$\begin{array}{c} q\left(\beta ,V\right)=\frac{1}{Q^{N}}\int_{r_{1}>d}d^{3}r_{1}\cdots \int_{r_{N}>d}d^{3}r_{N}\;\;\prod_{\left(ij\right)}\left(1+f_{ij}\right)\;\\ =\frac{1}{Q^{N}}\int_{r_{1}>d}d^{3}r_{1}\cdots \int_{r_{N}>d}d^{3}r_{N}\;\;\left(1+\sum_{ij}f_{ij}+\sum_{ij}\sum_{kl}f_{ij}f_{kl}+\cdots \right). \end{array}$$

Assuming that the parameter $\phi_{0}$ in the economic potential is sufficiently small, the contribution arising from $f_{ij}$ in the specified integration range can be regarded as an infinitesimal. The first term on the right side of (8), i.e., the integral of unity, can, on the other hand, be approximated by

$$Q\left(Q-Q_{0}\right)\cdots \left[Q-\left(N-2\right)Q_{0}\right]\left[Q-\left(N-1\right)Q_{0}\right]\approx {\left(Q-bN\right)}^{N},$$

where we put $Q_{0}=2b=\frac{4}{3}\pi d^{3}$. The integrations are performed consecutively, so that the first actor can occupy the volume of goods *Q* without constraints, the second actor can occupy the volume of goods *Q* less the volume $Q_{0}$ occupied by the first actor, the third actor can occupy volume of goods *Q* less the volume $2Q_{0}$ occupied by the first two actors, and so on. Similarly, the second term on the right side of (8) can be approximated by

$$\begin{array}{c} \int_{r_{1}>d}d^{3}r_{1}\cdots \int_{r_{N}>d}d^{3}r_{N}\;\;f_{ij}={\left(Q-bN\right)}^{N-1}\int_{r_{j}>d}f_{ij}\;\\ \approx {\left(Q-bN\right)}^{N-1}\beta \pi \int_{d}^{\infty }\phi \left(r\right)r^{2}dr. \end{array}$$

Assembling these results, we can approximate q(β,Q) in the following closed form

$$\begin{array}{c} q\left(\beta ,Q\right)\approx {\left(1-b\;\;\frac{N}{Q}\right)}^{N}\left(1+\beta \;\;\frac{aN^{2}}{Q^{2}}+\cdots \right)\;\\ \approx {\left(1-b\;\;\frac{N}{Q}\right)}^{N}\exp\left(\beta \;\;\frac{aN^{2}}{Q^{2}}\right), \end{array}$$

where we have defined $a=-\pi \int_{d}^{\infty }\phi \left(r\right)r^{2}dr$. Using the above expression for q(β,Q), we finally obtain the canonical partition function

(9) $$z\left(\beta ,Q\right)=\frac{1}{N!}{\left(\frac{2\pi y}{\beta h^{2}}\right)}^{3N/2}{\left(Q-bN\right)}^{N}\exp\left(a\beta N^{2}/Q\right).$$

From the expression for the partition function of the canonical distribution, we deduce the equation of state

$$P=\frac{1}{\beta }{\left(\frac{\partial }{\partial Q}\ln z\left(\beta ,Q\right)\right)}_{\beta }=\frac{Nk_{B}I}{Q-bN}-a\;\;\frac{N^{2}}{Q^{2}},$$

where for clarity we have substituted $\beta =1/k_{B}I$. Observe that this is precisely the Van der Waals equation. If we had not applied various approximations in the derivation of (9), then additional terms of order ${\left(N/Q\right)}^{3}$ and higher would have appeared on the right side of the previous formula. Similarly, the Gibbs free energy is

$$G=PQ-k_{B}I\;\;\ln z\left(I,Q\right).$$

### 3.3. The Economic Limit

The existence of the instability in the economic Van der Waals system studied above is related to the fact that, in the canonical distribution, the volume of goods *Q* of the system is held fixed, whereas, in a real income, volume fluctuations are significant in the neighborhood of the critical point. In other words, the canonical distribution does not provide a completely accurate economic description of the “income—monetary policy of liquidity” equilibrium. Therefore, as in the case of an ideal income, we consider the “price level—internal politics stability” distribution, with the corresponding partition function

(10) $$Z\left(\alpha ,\beta \right)=\frac{1}{b}\int_{bN}^{\infty }z\left(\beta ,Q\right)\;\;\exp\left(-\alpha Q\right)\;\;dQ,$$

wherein the volume of goods fluctuation is integrated out. Recall that $b=\frac{2}{3}\pi d^{3}$ represents the smallest volume each actor can occupy. Hence, the random variable *Q* representing the total volume of goods ranges from bN to infinity. When the canonical partition function (9) is substituted into (10), the resulting integral does not admit an elementary analytical expression. Nevertheless, in the thermodynamic limit N→∞, we can implicitly determine the potential $\psi \left(\alpha ,\beta \right)=\lim_{N\to \infty }N^{-1}\ln Z\left(\alpha ,\beta \right)$ by the method of steepest descent [22].

Changing the variable, $\hat{s}=N^{-1}\;\;Q$, the integral becomes

$$Z\left(\alpha ,\beta \right)=\frac{1}{b}\int_{b}^{\infty }z\left(\beta ,N\hat{s}\right)\;\;\exp\left(-\alpha N\hat{s}\right)\;\;d\hat{s}.$$

The method of steepest descent requires us to write the integrand under exponential special form

$$z\left(\beta ,N\hat{s}\right)\;\;\exp\left(-\alpha N\hat{s}\right)=\exp\left[Ng\left(\alpha ,\beta ,\hat{s}\right)\right],$$

where $\hat{s}=N^{-1}\;\;Q$ and

$$\begin{array}{c} g\left(\alpha ,\beta ,\hat{s}\right)=N^{-1}\left(\ln z\left(\beta ,N\hat{s}\right)-\alpha \hat{s}\right)\;\\ =1-\alpha \hat{s}+\ln\left[{\left(\frac{2\pi y}{\beta h^{2}}\right)}^{3/2}\left(\hat{s}-b\right)\right]+\frac{\beta a}{\hat{s}}, \end{array}$$

via Stirling formula lnN!≈NlnN−N. It should be evident that the Gibbs free energy is now

$$G=-\beta^{-1}g\left(\alpha ,\beta ,\hat{s}\right).$$

Recall that we are interested in the economic potential per actor as the economic limit

$$\psi \left(\alpha ,\beta \right)=\lim_{N\to \infty }N^{-1}\ln Z\left(\alpha ,\beta \right).$$

Let $\bar{s}=\bar{s}\left(\alpha ,\beta \right)$ be the function of α and β which maximises $g(\alpha ,\beta ,\hat{s})$. Then, using the method of steepest descent, we find that ψ(α,β) in the previous limit is given by

$$\psi \left(\alpha ,\beta \right)=g\left(\alpha ,\beta ,\bar{s}\right)=-\alpha \bar{s}+\ln z\left(\beta ,\bar{s}\right).$$

However, in the P−I distribution, the volume of goods is a random variable, hence we must take its expectation to obtain the equation of state

$$\langle \hat{s}\rangle =-\frac{1}{N}\frac{\partial }{\partial \alpha }\;\;\ln Z\left(\alpha ,\beta \right),$$

where $\langle \hat{s}\rangle$ denotes the expected volume per particle in the P−I distribution characterised by the density function $Z^{-1}\left(\alpha ,\beta \right)\exp\left[Ng\left(\alpha ,\beta ,\hat{s}\right)\right]$.

Since $\bar{s}=\bar{s}\left(\alpha ,\beta \right)$ minimises the Gibbs free energy, it is the solution of the economic Van der Waals equation of state. Although the exact form of function $\bar{s}\left(\alpha ,\beta \right)$ is not at our disposal owing to the cubic nature of the equation of state, we can nonetheless determine the exact expression for the scalar curvature in terms of the variables β and $\bar{s}$. Before we proceed, however, we first establish the following result

**Proposition** **3.** *The economic expectation value of the volume of goods $\hat{s}$ per actor in the P−I distribution is given by $\bar{s}$, i.e., $\langle \hat{s}\rangle =\bar{s}$.*

**Proof.**  We compute the partial derivative

$$\frac{\partial \psi }{\partial \alpha }=-\bar{s}+\left(-\alpha +\frac{\partial \ln z}{\partial \bar{s}}\right)\frac{\partial \bar{s}}{\partial \alpha }.$$

Since $\bar{s}$ is a maximum point of $g(\alpha ,\beta ,\hat{s})$, we must have

(11) $$\frac{\partial g}{\partial \bar{s}}=-\alpha +\frac{\partial \ln z}{\partial \bar{s}}=0$$

and hence $\frac{\partial \psi }{\partial \alpha }=-\bar{s}$. On the other hand, from a previous formula, we have $\langle \hat{s}\rangle =-\frac{\partial \psi }{\partial \alpha }$, and thus $\langle \hat{s}\rangle =\bar{s}$. □

### 3.4. Geometry of the Economic Van Der Waals Manifold

Let us find the Fisher–Rao geometry on the Van der Waals manifold M.

As we have just indicated, the function $\bar{s}=\bar{s}\left(\alpha ,\beta \right)$ is only implicitly known. Consequently, we can determine the expressions for the partial derivatives $\frac{\partial \bar{s}}{\partial \alpha }$, $\frac{\partial \bar{s}}{\partial \beta }$ and so on via the implicit function theorem. To do that, we consider the equation

$$G\left(\alpha ,\beta ,\bar{s}\right)=-\frac{\partial g}{\partial \bar{s}}=\alpha -\frac{1}{\bar{s}-b}+\beta \;\;\frac{a}{{\bar{s}}^{2}}=0,$$

which defines the implicit function $\bar{s}=\bar{s}\left(\alpha ,\beta \right)>0$ (this is just the equation of state for the Van der Waals income). In general notations, the implicit function theorem gives

$$\frac{\partial \bar{s}}{\partial \alpha }=-\frac{\partial G}{\partial \alpha }/\frac{\partial G}{\partial \bar{s}},\;\frac{\partial \bar{s}}{\partial \beta }=-\frac{\partial G}{\partial \beta }/\frac{\partial G}{\partial \bar{s}}.$$

Automatically, we find

$$\frac{\partial G}{\partial \alpha }=1,\;\;\frac{\partial G}{\partial \beta }=\frac{a}{{\bar{s}}^{2}},\;\;\frac{\partial G}{\partial \bar{s}}=\frac{1}{{\left(\bar{s}-b\right)}^{2}}-\frac{2a}{{\bar{s}}^{3}}\;\;\beta .$$

Accepting $D=\frac{2a}{{\bar{s}}^{3}}\;\;\beta -\frac{1}{{\left(\bar{s}-b\right)}^{2}}\neq 0$, we deduce

$$\frac{\partial \bar{s}}{\partial \alpha }=\frac{1}{D},\;\;\frac{\partial \bar{s}}{\partial \beta }=\frac{1}{D}\frac{a}{{\bar{s}}^{2}}.$$

**Remark** **1.** *The function $\bar{s}$ is a solution of the PDE $\frac{\partial \bar{s}}{\partial \beta }=\frac{\partial \bar{s}}{\partial \alpha }\;\;\frac{a}{{\bar{s}}^{2}},$ but this equation is not useful at this moment.*

We observe that D=0 is the equation for the spinodal curve, which contains the critical point $\left(P_{c},Q_{c},I_{c}\right)$. On the other hand, for the positiveness of the Fisher–Rao metric, we need the condition D<0.

The derivatives of $\bar{s}\left(\alpha ,\beta \right)$ with respect to the parameters α and β are required in order to determine the covariant components of the Fisher–Rao Hessian metric [1,2]

$$g_{11}=\frac{\partial^{2}\psi }{\partial \alpha^{2}},\;\;g_{12}=\frac{\partial^{2}\psi }{\partial \alpha \partial \beta },\;\;g_{22}=\frac{\partial^{2}\psi }{\partial \beta^{2}}$$

on the economic Van der Waals manifold M. Specifically, we obtain the following

**Proposition** **4.** *In terms of the “price level—internal politics stability” coordinates (α,β), the Fisher–Rao metric on the Van der Waals manifold M has the components*

$$g_{11}=-\frac{1}{D},\;\;g_{12}=-\frac{1}{D}\frac{a}{{\bar{s}}^{2}},\;\;g_{22}=\frac{3}{2}\;\;\beta^{-2}-\frac{1}{D}\;\;\frac{a^{2}}{{\bar{s}}^{4}}$$

*and $g=\det\left(g_{ij}\right)=-\frac{3}{2D\beta^{2}}>0.$ In particular, in the ideal income limit, a→0 and b→0 (or in the limiting case a→0 and b≠0), the metric in this proposition reduces to the metric in formula (4) for the ideal income.*

**Proof.**  The components of the Fisher–Rao metric are $g_{ij}=\partial_{i}\partial_{j}\psi \left(\alpha ,\beta \right)$. In Proposition 3, we have established that $\frac{\partial \psi }{\partial \alpha }=-\bar{s}$, and, using (11), we find

$$\frac{\partial \psi }{\partial \beta }=-\alpha \frac{\partial \bar{s}}{\partial \beta }+\frac{\partial \ln z}{\partial \bar{s}}\frac{\partial \bar{s}}{\partial \beta }+\frac{\partial \ln z}{\partial \beta }=\frac{\partial \ln z}{\partial \beta }.$$

Therefore, we obtain

$$\frac{\partial^{2}\psi }{\partial \alpha^{2}}=-\frac{\partial \bar{s}}{\partial \alpha },\;\;\frac{\partial^{2}\psi }{\partial \alpha \partial \beta }=-\frac{\partial \bar{s}}{\partial \beta },\;\;\frac{\partial^{2}\psi }{\partial \beta^{2}}=\frac{\partial^{2}\ln z}{\partial \beta^{2}}+\frac{\partial^{2}\ln z}{\partial \beta \partial \bar{s}}\;\;\frac{\partial \bar{s}}{\partial \beta },$$

whence the desired expression for the metric follows from the formula (9) for the canonical partition function. In the ideal income limit a→0 and b→0, we have $D\to -\frac{1}{{\bar{s}}^{2}}$. However, from the ideal income equation of state, we have $\bar{s}=\alpha^{-1}$, hence we recover the results in Section 1. □

For surfaces immersed or submersed in $R^{3}$, the *scalar curvature* is twice the *Gaussian curvature*, and completely characterizes the curvature of a surface.

To compute the scalar curvature *R* of the economic Van der Waals manifold M, we need the partial derivatives

$${\bar{s}}_{\beta }=\frac{1}{D}\frac{a}{{\bar{s}}^{2}},\;\;D_{\alpha }=-\frac{2}{D}\left(\frac{3a\beta }{{\bar{s}}^{4}}-\frac{1}{{\left(\bar{s}-b\right)}^{3}}\right)$$

$$D_{\beta }=-\frac{2a}{D{\bar{s}}^{2}}\left(\frac{3a\beta }{{\bar{s}}^{4}}-\frac{1}{{\left(\bar{s}-b\right)}^{3}}-\frac{D}{\bar{s}}\right)=\frac{a}{{\bar{s}}^{2}}\;\;D_{\alpha }+\frac{2a}{{\bar{s}}^{3}}.$$

**Proposition** **5.** *The scalar curvature R of the economic Van der Waals manifold M is*

$$R=\frac{9a}{2D^{6}{\bar{s}}^{8}\beta^{7}}\left(-\frac{8a^{3}}{D{\bar{s}}^{2}}\;\;\beta^{3}+6a{\bar{s}}^{2}\;\;\beta -3D{\bar{s}}^{5}\right).$$

**Proof.**  Generally, in an *n*-dimensional Riemannian manifold with a Hessian metric $g_{ij}=f_{ij}=\partial_{i}\partial_{j}f$, we have

$$\Gamma_{ijk}=\frac{1}{2}\;\;f_{ijk},\;\;R_{ijkl}=-\frac{1}{4}\;\;g^{pq}\left(f_{jlp}f_{ikq}-f_{ilp}f_{jkq}\right),\;\;R=g^{ik}g^{jl}R_{ijkl},$$

where $f_{ijk}=\partial_{i}\partial_{j}\partial_{k}f$. It is remarkable that the curvature tensor field of a Hessian metric depends only on the derivatives of *f* to order at most three, whereas one would expect fourth derivatives of *f* to come in.

Our statistical manifold M is two-dimensional. Then, the expression for the scalar curvature *R* admits a more simple form: denoting $\psi_{ij}=g_{ij}=\partial_{i}\partial_{j}\psi \left(\alpha ,\beta \right)$, $\psi_{ijk}=\partial_{i}\partial_{j}\partial_{k}\psi \left(\alpha ,\beta \right)$, we find the formula

$$R=-\frac{1}{2g^{2}}\left|\begin{array}{ccc} g_{11} & g_{12} & g_{22}\\ \psi_{111} & \psi_{112} & \psi_{122}\\ \psi_{112} & \psi_{122} & \psi_{222} \end{array}\right|.$$

Computing,

$$\psi_{111}=\partial_{1}g_{11}=\frac{1}{D^{2}}\;\;\partial_{1}D,\;\;\psi_{112}=\partial_{2}g_{11}=\frac{1}{D^{2}}\;\;\left(\frac{a}{{\bar{s}}^{2}}\;\;\partial_{1}D+\frac{2a}{{\bar{s}}^{3}}\right),$$

$$\psi_{122}=\partial_{2}g_{12}=\frac{a}{D^{2}{\bar{s}}^{2}}\;\;\left(\frac{a}{{\bar{s}}^{2}}\;\;\partial_{1}D+\frac{2a}{{\bar{s}}^{3}}\right)+\frac{2a}{D{\bar{s}}^{3}}\;\;\partial_{2}\bar{s},$$

$$\psi_{222}=\partial_{2}g_{22}=-\frac{3}{\beta^{3}}+\frac{a^{2}}{D^{2}{\bar{s}}^{4}}\;\;\left(\frac{a}{{\bar{s}}^{2}}\;\;\partial_{1}D+\frac{2a}{{\bar{s}}^{3}}\right)+\frac{4a^{2}}{D{\bar{s}}^{5}}\;\;\partial_{2}\bar{s},$$

and using a little algebra, we find our statement. □

**Corollary** **1.** *(1) R>0, and $\lim_{D\to 0}\;\;R=\infty$ (diverges along the entire spinodal curve); (2) the scalar curvature vanishes in the ideal income limit obtained from a→0 and b→0; but the scalar curvature vanishes also in the limiting case a→0 and b≠0.*

**Remark** **2** (see [24])**.** *The manifold M is not compact. We introduce the universal covering $\bar{M}$, which is compact.*

*Let M be a compact manifold with dimension 2. In this case, the scalar curvature R(x) coincides with the Gaussian curvature and the Gauss–Bonnet formula*

$$\chi \left(M\right)={\left(4\pi \right)}^{-1}\int_{M}R\left(x\right)\;\;dvol\left(x\right)$$

*relates it to the Euler–Poincaré characteristic χ(M), which is a topological invariant of the 2-manifold M.*

*Generally, if a 2-dimensional compact manifold M admits a metric of positive scalar curvature, then χ(M)>0 and, by the classification theorem of 2-manifolds, this implies that $M=S^{2}$ or $M=RP^{2}$ and, indeed, these manifolds do admit metrics of positive scalar curvature. Thus, χ(M)>0 if and only if M admits a metric of positive scalar curvature.*

## 4. Conclusions

A brief historical review of Information Geometry can be found in paper [20]. This field was historically motivated by providing some differential-geometric structures to statistical models in order to create a better representation and understanding of statistics.

Our modelling includes an economic thermodynamic dictionary (morphism), an economic Hamiltonian, a Fisher–Rao Hessian metric, geodesics for ideal income, Van der Waals income in Roegenian economics, a canonical partition function, the economic limits, and geometry of Van der Waals manifold.
